# The Bioactive Compound Contents and Potential Protective Effects of Royal Jelly Protein Hydrolysates against DNA Oxidative Damage and LDL Oxidation

**DOI:** 10.3390/antiox10040580

**Published:** 2021-04-09

**Authors:** Shu-Hua Chiang, Kia-Min Yang, Shiann-Cherng Sheu, Chih-Wei Chen

**Affiliations:** 1 Department of Health and Creative Vegetarian Science, Fo Guang University, Yilan 26247, Taiwan; shchiang@mail.fgu.edu.tw; 2 Department of Hospitality Management, Mingdao University, Changhua 523, Taiwan; a9241128@mdu.edu.tw; 3 Bachelor Degree Program in Environment and Food Safety Laboratory Science, Chang Jung Christian University, Tainan 71101, Taiwan; scschem@mail.cjcu.edu.tw

**Keywords:** royal jelly, protein hydrolysate, DNA oxidative damage, LDL oxidation, conjugated diene, Fenton reaction

## Abstract

In this study, the inhibition of DNA oxidative damage and low-density lipoprotein (LDL) oxidation of royal jelly protein (RJP) hydrolysates obtained from two commercial proteases were investigated. The results showed that the inhibition of DNA oxidative damage induced by the Fenton reaction, RJP, RJPs hydrolyzed by alcalase (RJP-A), RJPs hydrolyzed by flavourzyme (RPJ-F) and RJP two-stage hydrolysates (RPJ-AF) all had the effect of inhibiting deoxyribose oxidative damage. The inhibition effect of RJP, RJP-A, RJP-F and RJP-AF (1.0 mg/mL) were 47.06%, 33.70%, 24.19% and 43.09%, respectively. In addition, studies have also found that both RJP and RJP hydrolysates can reduce the production of 8-OH-2′-dG and the order of its inhibitory ability is RJP-AF ≒ RJP-A > RJP-F > RJP. The inhibition of DNA damage induced by bleomycin-Fe^3+^/ascorbic acid (Asc) with the addition of RJP, RJP-A, RPJ-F and RPJ-AF were 17.16%, 30.88%, 25.00% and 37.25%, respectively. The results of LDL oxidation inhibition showed that RJP-AF (1 mg/mL) not only had the most effective inhibitory Cu^2+^-induced LDL oxidation to produce a thiobarbituric acid reactive substance (TBARS) but also extended the lag time of conjugated diene formation to 300 min, which was 3.3 times that of the control group.

## 1. Introduction

Functional foods contain health-promoting compounds as well as traditional nutrients [1]. The value of functional foods can be enhanced by adding bioactive peptides, polyunsaturated fatty acids, probiotics and/or prebiotics [2]. Protein hydrolysate is a mixture of peptides, free amino acids and other proteases. Protein hydrolysate products have different physiological activities based on enzyme specificity and the degree of hydrolysis (DH) [3]. Royal jelly (RJ) is an attractive ingredient in healthy foods and has for some time been in use commercially. It is also the sole food for the queen honeybee [4] and of the honeybee larva and is present in the secretions from the hypopharyngeal and mandibular glands of 2 to 12 day old adult workers [5,6,7]. RJ contains 12–15% protein, of which 83–90% belongs to the major royal jelly protein (MRJP) family [8,9]. The pharmacological and biological functions of MRJPs include inhibiting cell proliferation [10], suppressing cytokine production [11] and antitumor [12], antioxidant [13], immunostimulatory [7,12], hepatoprotective [14] and antimicrobial activities [15]. RJ protein (RJP)-induced activities are widely useful in maintaining homeostasis and recovery from pathological conditions; therefore, RJ has been used in cosmetics, health food or as a dietary supplement [16].

In a living cell, DNA is a repository of genetic information and is remarkably susceptible to damage induced by exogenous and endogenous factors [3,17,18]. Reactions that cause DNA damage include oxidation, methylation and deamination [19], among which oxidation is highly significant. DNA damage caused by reactive oxygen species (ROS) such as hydroxyl radicals (^●^OH), hydrogen peroxide (H_2_O_2_) and superoxide (O_2_^−^) leads to cell repair or failure to repair the errors caused by the split mutations [3,18]. Additionally, ROS directly interfere with cell signal transmissions and the initiation of mitosis; the increase in the degree of DNA damage further leads to mutations or increased exposure to the mutant DNA [18,20]. Therefore, the ROS scavenging capacity of antioxidants has been widely investigated in the food technology and human health fields [3,17,18].

Oxidation-modified low-density lipoprotein (LDL) is one of the main inducers of oxidative stress and a potential cause of coronary artery disease and strokes [21]. Within the arterial wall, oxidized LDL promotes atherosclerosis, leading to cardiovascular disease [3,22,23]. Epidemiologic studies have shown that dietary antioxidants contribute to the prevention of coronary heart disease [23]. Many antioxidants inhibit lipid oxidation although they fail to protect matrices such as DNA, carbohydrates and proteins from oxidative damage [24]. Therefore, it is essential to search for a bioavailable antioxidant to clear up radicals and to inhibit the oxidation of DNA in the human body [3,18].

Food protein hydrolysates are widely accepted and used in cosmetics and healthcare products. RJP is well known to enhance human health; however, RJP hydrolysates have not been examined for their inhibitory effect on cell DNA damage and LDL oxidation. In this study, RJP was hydrolyzed by alcalase and flavourzyme through one- and two-stage processes and the inhibition of cellular DNA oxidative damage and LDL oxidation were investigated.

## 2. Materials and Methods

### 2.1. Materials and Reagents

Fresh royal jelly (RJ) from *Apis mellifera* L. was obtained from the Honey Bee Farm (Changhua, Taiwan). Fresh RJ was dissolved in 0.01 M phosphate buffered saline (pH 7.2) and heated in a water bath (90 °C) for 30 min to eliminate its enzymatic activity. It was then lyophilized and stored at −20 °C until use.

The enzymes used for protein hydrolysis were alcalase (2.4 Anson units (AU)/g) and flavourzyme (0.5 unit/g) (Sigma Chemicals Co., St. Louis, MO, USA), respectively. TBA (2-thiobarbituric acid, minimum 98%), 8-hydroxy-2′-deoxyguanosine, 2′-deoxyguanosine monohydrate (2′-dG; 99–100%), bleomycin sulphate and calf thymus DNA were purchased from Sigma Chemicals Co. (St. Louis, MO, USA). All other chemicals and solvents used were of analytical grade.

### 2.2. Preparation of Different Protein Hydrolysates of Royal Jelly

The hydrolysis methods developed by Chiang et al. [3] and Wang and Chen [7] were summarized and adopted. One gram of RJ sample was dissolved in 100 mL of distilled water and stirred for 10 min at room temperature. The reaction pH levels were adjusted to 8.5 for alcalase hydrolysis (RJP-A) (E/S = 1.0, 1.5 and 2.0%) and 7.5 for flavourzyme hydrolysis (RJP-E) (E/S = 1.0, 1.5 and 2.0%) with 1 N NaOH or HCl. The enzymes were added to the mixture and allowed to react for 2, 4, 6, 8, 10, 12, 16, 20 and 24 h at 50 °C for alcalase and 40 °C for flavourzyme. The reactions were terminated by immersing the mixture into a water bath at 90 °C for 15 min. In the two-stage hydrolysis process, alcalase (E/S = 1.5%) was used in the first stage of hydrolysis for 4 h. Flavourzyme (E/S = 1.0, 1.5 and 2.0%) was used in the second stage and the hydrolysis durations were 2, 4, 6, 8, 10, 12, 16, 20 and 24 h. The two-stage hydrolysis (RJP-AF) was stopped by heat treatment at 90 °C for 15 min. The degree of hydrolysis (DH) of the RJP hydrolysates was calculated as (amino nitrogen/ total nitrogen) × 100%, where the total nitrogen and amino nitrogen contents were determined by the semi-micro-Kjeldahl method and the formol titration method Association of Official Analytical Chemists (AOAC) [25], respectively.

### 2.3. The (E)-10-Hydroxydec-2-Enoic Acid (10-HDA) Contents in RJP and RJP Hydrolysates

The 10-HDA content of different RJP samples was measured following the method described by Wang and Chen [7]. RJPs and their protein hydrolysates (0.25 g) were dissolved by sonication in 100 mL of a methanol solution (50:50 *v*/*v* with ultrapure water) then adjusted at pH = 2.5 with H_3_PO_4_. The sample solution (20 μL), previously diluted 10 times and filtered, was eluted on a RP-C18 column (Lichrospher 100, 5 μm, 250 × 4 mm, Merck, Darmstadt, Germany) with an aqueous solution of methanol as an eluant. The analysis was performed on Hitachi L-2200 liquid chromatography (Hitachi Co. Ltd., Tokyo, Japan) connected to a diode array detector (the detection wavelength was fixed at 210 nm). The 10-HDA content was determined by using a calibration curve based on standard solutions (1, 2, 5, 10, 20 and 40 μg/mL). The 10-HDA content (%) was then expressed as the ratio of the amount of 10-HDA detected to the number of different RJP samples weighed.

### 2.4. Flavonoids and Phenolic Acid Contents in RJP and RJP Hydrolysates

The flavonoids and phenolic acid content of different RJP samples were measured following the method described by Liao [26]. High performance liquid chromatography (HPLC) separation was done using an RP-18 GP250 column (L, 250 mm; ID, 4.6 mm; Kanto Chemical Co., Tokyo, Japan) maintained at room temperature with a mobile phase flow rate of 1.0 mL/min. The mobile phase contained solvent A (10% methanol with 0.05% formic acid) and solvent B (70% methanol with 0.05% formic acid); the maximum absorbance of flavonoids and phenolic acid was 280 nm. The injection volume was 10 μL. The identification and quantification were accomplished by comparing the retention time of peaks in the methanol containing solutions with those of the standard compounds.

### 2.5. Amino Acid Composition and Content in RJP and RJP Hydrolysates

A method developed by Wang and Chen [7] was utilized. All of the samples (100 mg for different RJP samples) were hydrolyzed with 10 mL of 6 M HCl at 110 °C for 22 h under a nitrogen atmosphere to obtain the total amino acids. The amino acid composition analysis used the Hitachi L-8900 amino acid analyzer (Hitachi Co. Ltd., Tokyo, Japan).

### 2.6. Effect of RJP and RJP Hydrolysates on the Damage to Deoxyribose (Fenton Reaction)

The degradation of deoxyribose was evaluated by the Fenton reaction that produces a thiobarbituric acid reactive substance (TBARS) [27]. The reaction mixture contained deoxyribose (3 mM), FeCl_3_ (50 μM), ascorbic acid (0.1 mM) and H_2_O_2_ (1 mM) in a KH_2_PO_4_ buffer (20 mM, pH 7.4) and various concentrations of RJP and RJP hydrolysates. The final reaction mixtures were incubated at 37 °C for 30 min. Following this, 1 mL TBA (1%) and 1 mL trichloroacetic acid (TCA; 2.8%) were added and heated at 100 °C for 20 min. The TBARS content was then measured as previously described by reading the absorbance at 532 nm.

### 2.7. Effect of RJP and RJP Hydrolysates on 2′-Deoxyguanosine (2′-dG) Oxidation (Fenton Reaction)

The effects of RJP and RJP hydrolysates on the oxidation of 2′-dG to 8-hydroxy-2′-deoxyguanosine (8-OH-2′-dG) were assayed using the method of Chen et al. [18] with modifications. The reaction containing the mixture (1.4 mL) ofRJP, RJP-A, RJP-F and RJP-AF samples (0.1 to 1.0 mg/mL), 2′-dG (0.5 mM) and a potassium phosphate buffer (20 mM, pH 7.4) wasinitiated using the Fenton reaction model system (H_2_O_2_, 50 mM; FeCl_3_, 1.3 mM; ethylenediaminetetraacetic acid (EDTA), 6.5 mM) with the addition of ascorbic acid (15 mM). The entire mixture was incubated at 37 °C for 30 min and incubation was terminated by placing the samples in an ice bath and then filtered through a 0.45 μm filter before use. The filtrate was analyzed by HPLC (Hitachi, Tokyo, Japan), using a LiChrosphere RP-18 column (150 mm × 4 mm, 5 μm; Merck, Darmstadt, Germany) and a UV detector (254 nm, Hitachi Co. Ltd., Tokyo, Japan). The mobile phase contained 6.5% ethanol in a 50 mM phosphate buffer and the flow rate was 0.5 mL/min. 2′-dG and 8-OH-2′-dG were identified by comparing their retention times with those of known standards and the amount of 8-OH-2′-dG was determined based on the peak areas in the chromatograms.

### 2.8. Effect of RJP and RJP Hydrolysates on Bleomycin-Dependent DNA Damage

The effect of RJP and RJP hydrolysates on bleomycin-dependent DNA damage was determined according to the method of Aruoma et al. [28]. The reaction mixtures (made up to 3.5 mL with phosphate buffered saline (PBS) contained calf thymus DNA (0.2 mg/mL), bleomycin (0.05 mg/mL), FeCl_3_ (25 μM) and MgCl_2_ (5 mM). Different concentrations of RJP and RJP hydrolysate (1, 2, 4, 6 and 10 μg/mL) were incubated for 1 h at 37 °C with or without the addition of ascorbic acid (240 μM). EDTA (0.1 mL; 100 mM) was added to the mixture, which was then measured using the TBA method as described above for the assay of deoxyribose damage.

### 2.9. Inhibition of Oxidative Damage of Biomolecules by RJP and RJP Hydrolysates

Two experiments were designed to assess the effect of RJP and RJP hydrolysates on the inhibition of oxidative damage of biomolecules in this study. One was assay estimated 2′-dG oxidation induced by the Fenton reaction and the other was estimated DNA oxidation induced by bleomycin-Fe^3+^ (1.5 mM) and ascorbic acid (10 μg/mL), which were added to the reaction solutions as specified in Section 2.7 and Section 2.8, respectively, as the simulators of oxidative damage. RJP and RJP hydrolysates (10 μg/mL) were then added to slow down the oxidation reaction. The resulting solutions were then analyzed at the end of the reaction using the procedures described in Section 2.7 and Section 2.8.

### 2.10. LDL Preparation and Oxidation

The LDL was collected and provided by Changhua Christian Hospital (Changhua, Taiwan). Briefly, 2 mL of LDL and 10 mM phosphate buffered saline (pH 7.4) were pipetted into an activated cellulose dialysis tube. The sealed tube was immersed in a beaker containing 10 mM PBS and dialyzed in the dark at 4 °C for 24 h. The protein concentration was measured by the Lowry method [29] and adjusted to 100 μg protein/mL before use. The oxidative stresson the LDL was caused by adding 40 μL of 2 mM copper sulphate (CuSO_4_) and incubated at 37 °C for 24 h. The reaction mixture without the sample extract was considered to be the control. The level of LDL oxidation was studied by both conjugated dienes (CD) and Thiobarbituric Acid Reactive Substance (TBARS) assay. The ascorbic acid oxidation was used for reference.

#### 2.10.1. Estimation of the Thiobarbituric Acid Reactive Substance (TBARS)

After incubation, the oxidation reaction was stopped using EDTA (1.5 mg/mL). A sample (0.2 mL) was mixed with 0.2 mL (20% *w*/*v*; pH 3.5) TCA and 0.2 mL (0.78% *w*/*v*) TBA solution and incubated for 45 min at 90 °C. This was then centrifuged (2000× *g*, 5 min) and fluorescence (λexc = 532 nm, λem = 600 nm, Hitachi F-3010, Hitachi Co. Ltd., Tokyo, Japan) was measured. As a standard, 1,1,3,3-tetramethoxypropane was used for the calibration curve and the content of the TBARS was calculated.

#### 2.10.2. Conjugated Diene Evolution

During incubation, the capacity of RJP and RJP hydrolysates to prevent LDL oxidation was evaluated by monitoring the generation of a conjugated diene. The absorbance was read every 30 min for 540 min using a Hitachi U-2000 spectrophotometer (Hitachi Co. Ltd., Tokyo, Japan) and the results were expressed as a relative absorbance at 234 nm. For each assay, measurements were performed at least in triplicate. The duration of the lag phase was calculated by extrapolating the exponential phase.

### 2.11. Statistical Analysis

All values are presented as the mean ± standard deviation (SD). Data were analyzed using a one-way analysis of variance and Duncan’s multiple range test. All statistical analyses were performed using SAS software (version 9.1, SAS Institute, Cary, NC, USA). The statistical significance was set at *p* < 0.05.

## 3. Results

### 3.1. 10-HDA, Flavonoids and Phenolic Acid Contents in RJP and RJP Hydrolysates

Table 1 shows that the 10-HDA contents of RJP and RJP hydrolysate varied between 2.32% and 2.95%. The 10-HDA content of RJP-AF was the highest (2.95 ± 0.01 mg/100 mg) and that of RJP was lowest (2.32 ± 0.03 mg/100 mg). The decreasing sequence of the 10-HDA contents of RJP and RJP hydrolysates was RJP-AF > RJP-A ≒ RJP-F > RJP.

The flavonoids and phenolic acid contents in RJP and RJP hydrolysates are also shown in Table 1. The quercetin, naringin and galangin contents of RJP-AF were the highest (18.44, 0.76 and 0.57 mg/100 mg, respectively) than the others; the naringin and hesperetin contents of RJP were the lowest (0.47 and 0.85 mg/100 mg, respectively). The phenolic acid content in RJP hydrolysates did not show a significant difference (Table 1). The chlorogenic acid contents in RJP and RJP hydrolysates were 37.61, 40.33, 38.26 and 39.68 mg/100 mg, respectively; the caffeic acid contents were 5.14, 4.76, 4.89 and 5.06 mg/100 mg, respectively; the ferulic acid contents were 68.42, 72.54, 74.31 and 73.22 mg/100 mg, respectively.

### 3.2. Degree of Hydrolysis (DH) of RJ Protein

**Figure 1:**
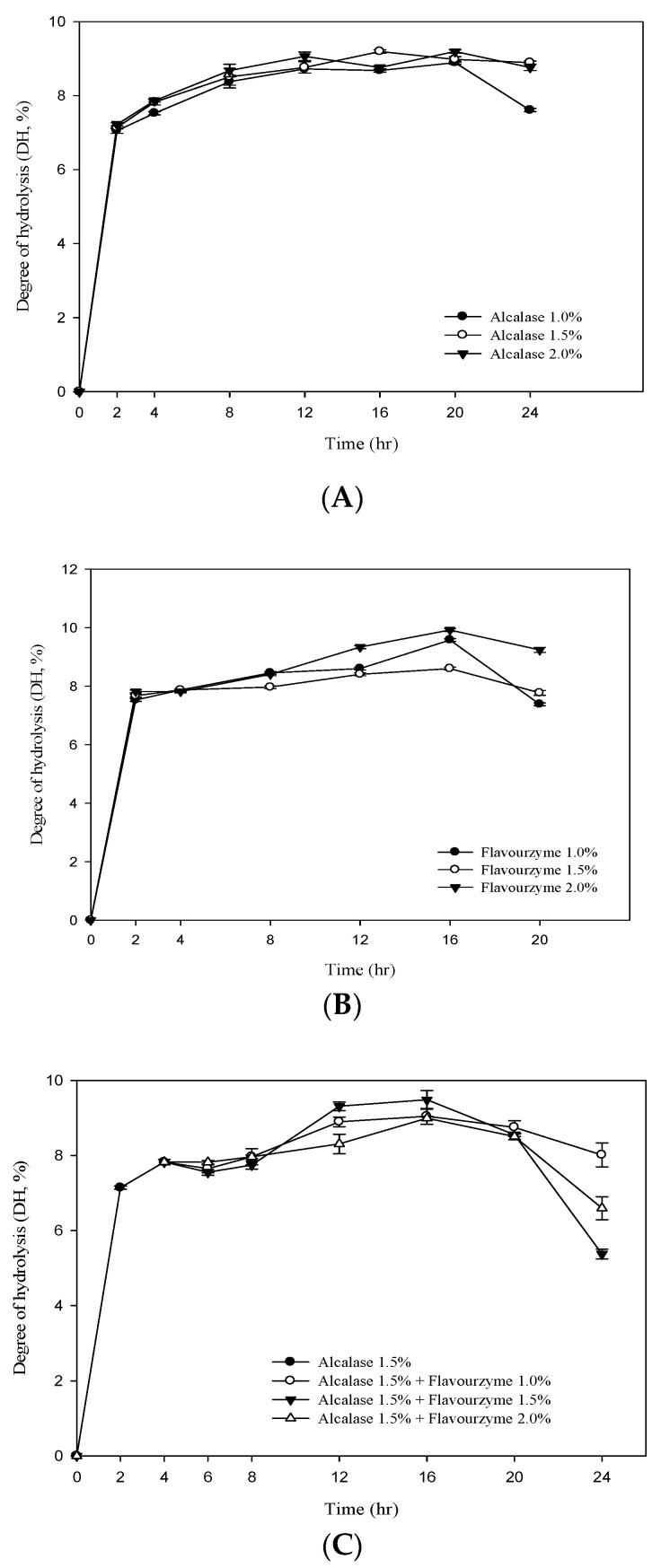
Degree of hydrolysis (DH%) of royal jelly protein hydrolyzed (**A**) by alcalase; (**B**) by flavourzyme and (**C**) during a two-stage hydrolysis using alcalase and flavourzyme.

Figure 1A,B shows the DH value of RJ protein by adding different concentrations ofalcalase and flavourzyme(enzyme/substrate ratios = 1%, 1.5% and 2.0%). The results showed that the DH value of RJ protein increased significantly with the increase in the assay time. At the beginning of the hydrolysis (2 h), the DH values (E/S = 1.5) of the two enzymes were 7.13% and 7.67% foralcalase and flavourzyme, respectively. After 20 h of hydrolysis, the DH values of the two (E/S = 1.5) were 8.99% and 8.59%, respectively. The two-stage hydrolysis of RJ protein by alcalase and flavourzyme was monitored for up to 20 h (Figure 1C). In the first stage, the highest DH value was 7.82% using alcalase (hydrolysis time 4 h; E/S = 1.5%). In the second stage, flavourzyme was added (hydrolysis time 16 h; E/S = 1.0%, 1.5% and 2.0%) and the DH values were 9.03%, 9.47% and 8.98%. Figure 1C clearly indicates that the DH increased with an increasing enzyme concentration and that it was positively related to the duration of hydrolysis. The study also found that the DH tended to decrease when the hydrolysis time was between 16–24 h (Figure 1). The reason may be the plastein effect reaction. The plastein reaction refers to the formation of gel-like or plastein-type structures on incubation of high concentrations of protein hydrolysates with proteinases. The plastein reaction recombines the protein, thus causing the DH to decrease.

### 3.3. The Amino Acid Compositions and Content of RJP and RJP Hydrolysates

The total amino acid compositions of RJP and RJP hydrolysates are shown in Table 2. The results showed that the RJP-AF contained the most necessary amino acids including the basic amino acid (BAA), His (94.73 nmole/mL), Arg (105.22 nmole/mL), Lys (487.93 nmole/mL); the branched chain amino acid (BCAA), Val (48.27 nmole/mL), Leu (102.48 nmole/mL), Ile (128.49 nmole/mL); aromatic amino acids such as Tyr (81.26 nmole/mL), Phe (93.67 nmole/mL) and other amino acids that are beneficial to the human body such as the acidic amino acid: Asp (36.88 nmole/mL), Glu (109.67 nmole/mL); the Thio group (-SH), Cys (65.58 nmole/mL), Met (113.88 nmole/mL) and the Hydroxy group (-OH), Ser (224.73 nmole/mL), Thr (46.92 nmole/mL) and Pro (95.93 nmole/mL). In addition, the necessary amino acid contents of RJP, RJP-A, RJP-F and RJP-AF were 203.32, 1115.87, 1032.74 and 1129.59 nmole/mL, respectively.

### 3.4. Effect of RJP and RJP Hydrolysates on the Fenton Reaction-Induced Oxidative Damage of Deoxyribose

**Figure 2:**
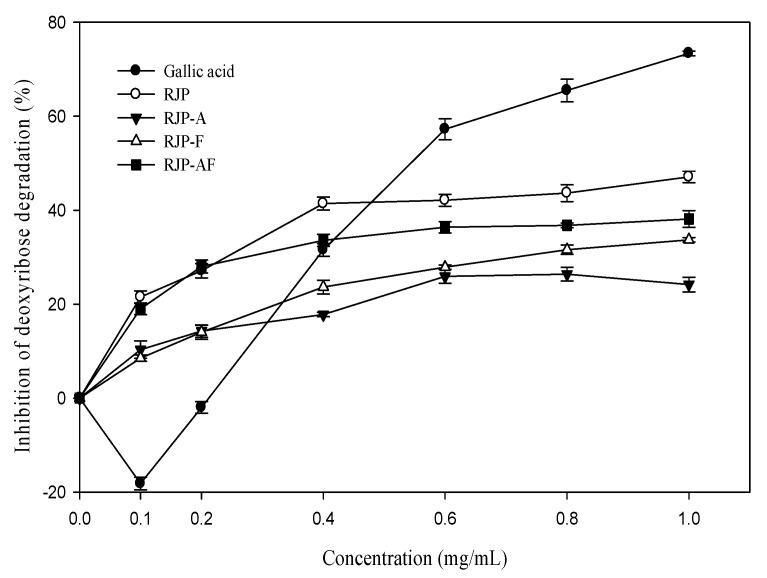
Effect of RJP and RJP hydrolysate on the Fe^2+^-EDTA/H_2_O_2_/ascorbic acid (Asc)-induced oxidative damage of deoxyribose. RJP: Royal jelly protein, RJP-A: Royal jelly protein hydrolyzed by alcalase, RJP-F: Royal jelly protein hydrolyzed by flavourzyme, RJP-AF: Royal jelly protein hydrolyzed by alcalase followed by flavourzyme.

Figure 2 shows that RJP and RJP hydrolysates had an inhibitory effect on the oxidation damage of deoxyribose induced by the Fe^3+^-EDTA/H_2_O_2_/Asc system. The inhibitory effect was concentration-dependent. The inhibitory effect of RJP was 21.49%at 0.1 mg/mL and 41.27% at 1.0 mg/mL, respectively. Figure 2 also shows that the inhibitory effects of RJP hydrolysates (RJP-A, RJP-F and RJP-AF) were lower than that of RJP. The inhibition rates of RJP, RJP-A, RJP-F and RJP-AF were 41.27, 32.74, 26.81 and 21.03% at 1 mg/mL, respectively. In contrast, gallic acid showed a concentration-dependent biphasic effect and exhibited a pro-oxidant effect at a low concentration but exhibited an antioxidant effect with an increasing concentration and reached 75% inhibition at 1.0 mg/mL.

### 3.5. Effect of RJP and RJP Hydrolysateson the Oxidation of 2′-Deoxyguanosine (2′-dG) to 8-Hydroxy-2′-Deoxyguanosine (8-OH-2′-dG) Induced by the Fenton Reaction

As the results showed (Table 3), at 0.125 mg/mL, RJP and RJP-F reduced 8-OH-2′-dG to 0.103 μg and 0.009 μg, respectively, compared with the control (0.224 μg). At the same concentration, RJP-A and RJP-AF completely inhibited the formation of 8-OH-2′-dG. However, ascorbic acid improved the formation of 8-OH-2′-dG (3.41 μg). Compared with ascorbic acid and the control, the sample solutions did not promote the generation of 8-OH-2′-dG under any circumstances. The hydrolysates (RJP-A, RJP-A and RJP-AF) exhibited better protective effects than RJP.

### 3.6. Effect of RJP and RJP Hydrolysates on Bleomycin-Dependent DNA Damage

**Figure 3:**
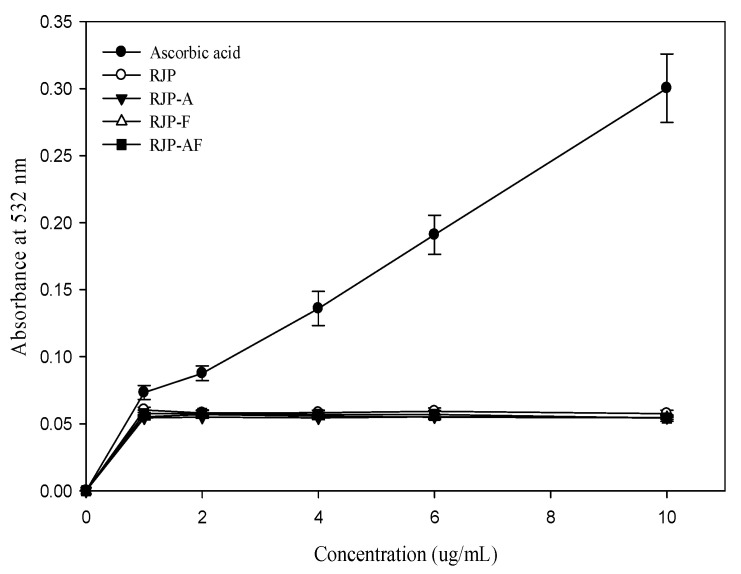
Effect of RJP and RJP hydrolysate on DNA damage induced by bleomycin-Fe^3+^.

The effect of RJP and RJP hydrolysates on DNA damage induced by bleomycin-Fe^3+^ is illustrated in Figure 3. Ascorbic acid was found to exert a pro-oxidant effect. In contrast, the pro-oxidant effect of RJP and RJP hydrolysates were not significant. Thus, RJP and RJP hydrolysates did not promote DNA damage induced by bleomycin-Fe^3+^.

### 3.7. The Protective Effects and Inhibition of Oxidative Damages of Biomolecules by RJP and RJP Hydrolysates

The addition of RJP and RJP hydrolysates in the initial stage of the reaction significantly reduced the oxidative damage caused by the addition of ascorbic acid in the subsequent stage (Table 4). The protective effect of RJP-AF was 80.97% and the protective effect was in the order of RJP-AF > RJP-A ≒ RJP-F > RJP (Table 2). Based on the results of 8-OH-2′-dG generation and DNA oxidative damage caused by the bleomycin-Fe^3+^ system, ascorbic acid may cause obvious oxidative damage due to the presence of Fe^3+^. Considering the effects of RJP and RJP hydrolysates on bleomycin-Fe^3+^/Asc-induced oxidative damage of DNA, the rates of inhibition of oxidative damage were 17.16, 30.88, 25.0 and 37.5%, respectively (Table 4). When using RJP and RJP hydrolysates on Fe^2+^-EDTA/H_2_O_2_/Asc-induced 2′-dG to produce 8-OH-2′-dG, the inhibition rates were 33.01, 48.38, 43.87 and 55.24%, respectively.

### 3.8. Effect of RJP and RJP Hydrolysates on the Formation of a Thiobarbituric Acid Reactive Substance (TBARS) and Conjugated Diene Formation by LDL Oxidation Induced by Cu^2+^

**Figure 4:**
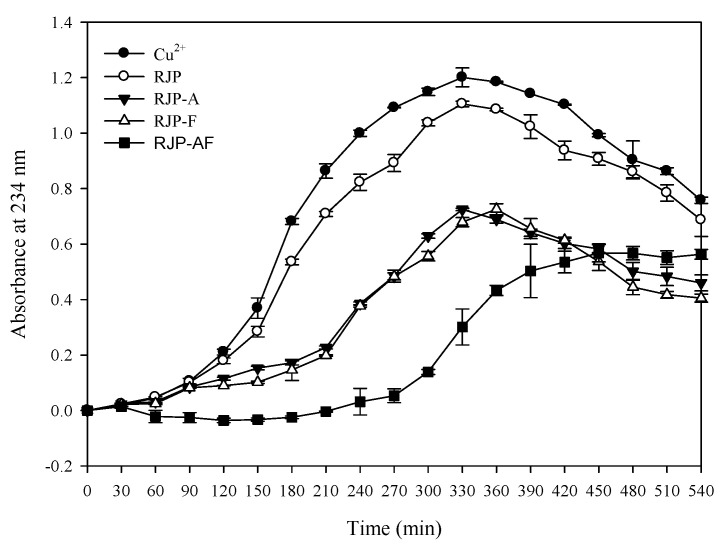
Effects of RJP and RJP hydrolysates on Cu^2+^ mediated conjugated diene formation in low-density lipoprotein (LDL).

Table 5 shows that the amount of the TBARS in the control group was 5.35 nM/mL. When the concentration was 1 mg/mL, the TBARS output was 4.26, 3.64, 3.75 and 3.28 nM/mL through the RJP, RJP-A, RJP-F and RJP-AF treatment, respectively. The amount of the TBARS in the RJP and RJP hydrolysates groups was lower than in the control group and the inhibitory effect of the TBARS formed by RJP-AF was better than that from the other three. Table 3 and Figure 4 show the effects of RJP and RJP hydrolysates on the oxidation of LDL induced by Cu^2+^ to form conjugated dienes. In the control group, only LDL and Cu^2+^ were added and the lag time was 90 min. When the concentration was 0.01 mg/mL, the lag time of all groups was very close to that of the control group. When the concentration was 0.1 mg/mL, the lag time of the RJP hydrolysates was extended to 180–270 min (Table 5 and Figure 4) but we did not observe a significant extension in the lag time of the RJP group at the same concentration. When the concentration of the RJP group was 1.0 mg/mL, a significant inhibitory effect was observed. The lag times of RJP, RJP-A, RJP-F and RJP-AF were 150, 210, 210 and 300 min, respectively, and were 1.66, 2.33, 2.33 and 3.33 times that of the control group.

## 4. Discussion

Several studies have suggested the development of functional raw materials to improve physiological activity. For example, enzymatic treatments have been proven to be effective for improving the quality of certain existing raw materials by modifying their structure or redistributing their composition [30,31]. Various protein hydrolysates have been used in cosmetics and healthcare. The physiological function activities of protein hydrolysates depend on enzyme specificity and the DH [32,33]. Protein hydrolysates containing more free amino acids and peptides have been proven to have better biological activity than unhydrolyzed proteins. For functional peptides with a progressive hydrolysis reaction, the gradual decomposition of the protein into multi-peptides of a variety of molecular weights such as tri-peptides, dipeptides and amino acids increase its nutritional and functional effectiveness [34].

It is well known that flavonoids and phenolic compounds belong to the bioactive components of plant products and have good health-promoting activities [26]. In this study, we reported that RJP and RJP hydrolysates showed significant contents of flavonoids and phenolic acids (Table 1) and that RJP and RJP hydrolysates also showed significance against DNA oxidative damage and LDL oxidation (Table 3, Table 4 and Table 5; Figure 2, Figure 3 and Figure 4). 10-HDA is only found in RJ so it has been used as a quality marker of royal jelly products [35,36]. Several pharmacological activities such as the antioxidant activities of RJ have already been confirmed by animal experiments. The results in Table 1 show that the 10-HDA content in RJP and RJP hydrolysates were 2.32, 2.74, 2.68 and 2.95%, respectively. The RJP-AF had the highest content of 10-HDA and also showed the best antioxidant activities in this study. In addition, many studies have confirmed glutathione (GSH) has a good antioxidant capacity [37]; GSH is composed of Gly, Glu and Cys. It can be speculated from the results in Table 2 that RJP and RJP hydrolysates are rich in free amino acids and their antioxidant capacity may be related to the amino acids contained in them.

Deoxyribose breaks down into malondialdehyde (MDA) under the attack of hydroxyl free radicals. MDA leads to DNA oxidative damage [38]. The results of this study indicated that RJP and RJP hydrolysates did not promote any oxidation and were rather good hydroxyl radical scavengers (Figure 2). RJP and RJP hydrolysates were very good inhibitors of oxidation. This may be related to their weaker reduction power. Wang and Chen [7] showed that RJP and RJP hydrolysates possessed a strong ability to scavenge hydroxyl and DPPH free radicals and chelate ferrous ion but their reducing power was found to be weak. Although the reducing power plays an important role in oxidation stability, it is more important in promoting oxidation especially in the presence of transition metals. The reducing power may promote the oxidative damage of biomolecules [39]. A strong reducing power can reduce Fe^3+^ to Fe^2+^ and increase the formation of hydroxyl radicals [24]. RJP hydrolysate is also rich in free amino acids, making it a stronger reductant than RJP [7]. This may be a possible reason for a lower inhibitory effect of RJP hydrolysates than that of RJP.

An oxidative DNA product, 8-OH-2′-dG, formed by oxygen free radicals, has been determined frequently in both in vivo and in vitro studies as a DNA oxidative damage marker and is also a potential mutagen [3,18,38]. Therefore, the determination of the 8-OH-2′-dG content could be useful in evaluating the oxidative stress in the body [40]. According to the results in Table 1, the inhibitory activity of these samples was in the order RJP-AF ≒ RJP-A > RJP-F > RJP at 0.125 mg/mL although ascorbic acid presented a significant pro-oxidant activity (*p* < 0.05) compared with the control. This may be because RJP and RJP hydrolysates do not have a strong reducing power [17]. We observed that RJP and RJP hydrolysates inhibited oxidative damage in 2′-deoxyguanosine in a trend opposite to that of the deoxyribose system.

Bleomycin, an antitumor antibiotic, can bind to DNA and cause single-strand breaks in the presence of O_2_ and Fe^2+^ [41]. The presence of a reducing agent in the reaction system may accelerate the rate of bleomycin-Fe^3+^-induced DNA damage. A bleomycin assay has been used to assess pro-oxidant effects [42]. Accordingly, in this assay, bleomycin was used to assess the pro-oxidant activity of RJP and RJP hydrolysates. RJP and RJP hydrolysates did not exhibit any pro-oxidant effect (Figure 3), clearly explaining that RJP and RJP hydrolysates did not possess a strong reducing power and were not capable of reducing bleomycin-Fe^3+^ to bleomycin-Fe^2+^ and causing DNA damage.

Bleomycin-Fe^3+^/Asc was then used to evaluate the antioxidant potential of RJP and RJP hydrolysates. In the initial phase of the study, ascorbic acid was added to the reaction system and the oxidative damage was observed. RJP and RJP hydrolysates were then added and the inhibition rates were calculated and compared with that of ascorbic acid. Table 4 shows that irrespective of the experimental system, RJP and RJP hydrolysates had a better protective effect in the inhibition of DNA oxidative damage. The protective effect of RJP hydrolysates was found to be better than that of RJP (Table 4) and the best protective effect was that of RJP-AF. Chen et al. tested the effects of whey, casein and skimmed milk on Fe^2+^-EDTA/H_2_O_2_/Asc-induced 8-OH-2′-dG from 2′-dG and found that their inhibition rates were 20.01%, 18.18% and 11.07%, respectively [18]. Using the same system, Chiang et al. [3] observed that the inhibition rates of whey protein hydrolysate (WPH) and WPH fractions (>10 kDa and <10 kDa) were 28.68%, 30.46% and 30.76%. RJP hydrolysate was superior to RJP in 8-OH-2′-dG inhibition. Wang and Chen [7] reported that royal jelly protein has efficient antioxidant activities including Fe^2+^ chelation and free radical scavenging. Therefore, the reasons for the high inhibition rate of RJP hydrolysates include a high Fe^2+^ chelating activity and free radical scavenging.

We studied the effect of RJP and RJP hydrolysates on the formation of MDA by Cu^2+^ induced LDL oxidation. MDA easily participated in the nucleophilic addition reaction with TBA at a low pH and a high temperature. MDA is one of the constituents of a TBARS. The decrease in the TBARS production indicated that the sample could effectively inhibit LDL oxidation. Furthermore, compared with the control, RJP and RJP hydrolysates could reduce the formation of the TBARS (Table 5) indicating an effective inhibition of Cu^2+^-induced LDL oxidation by RJP and RJP hydrolysates. In addition, the amount of the TBARS generated by RJP hydrolysates was less than that by RJP. Thus, the ability to inhibit Cu^2+^-induced LDL oxidation was in the order of RJP-AF > RJP-A ≒ RJP-A > RJP. Table 3 also presented the effect of WPH and WPH fractions on Cu^2+^-induced LDL oxidation to form a conjugated diene. If the sample had an inhibitory effect on LDL oxidation, the lag time increased. The formation of a conjugated diene could be inhibited by treatment with RJP and RJP hydrolysates (Table 5 and Figure 4). The RJP-AF extended the lag time of the conjugated diene formation to 300 min, which was 3.3 times that of the control group. Chen et al. [18] and Chiang et al. [3] used bovine colostrum protein and WHP to study the inhibition of Cu^2+^-induced LDL oxidation in the formation of a conjugated diene. Both bovine colostrum protein and WHP could increase the lag time by 2.33 and 2.66 fold of the control group, respectively. These results indicated that RJP and RJP hydrolysates were efficient free radical scavengers and Fe^2+^ chelators, which not only prevented DNA oxidative damage but also inhibited LDL oxidation.

## 5. Conclusions

The royal jelly major protein (RJMP) has several biological functions such as antibacterial, antioxidant, anticancer and immunomodulatory activities [7,43,44]. However, there are few studies on the function of RJP hydrolysates. In this study, we showed that RJP and RJP hydrolysates not only were rich in bioactive compounds such as 10-HDA, flavonoids (quercetin, naringin and galangin), phenolic acids (chlorogenic acid, caffeic acid and ferulic acid) and necessary amino acids but also exerted a significant inhibitory effect on DNA oxidative damage in different biomolecules induced by the Fenton reaction and could inhibit LDL oxidation. However, the mechanism and other functional compositions such as the peptide sequence of RJP hydrolysates remain unclear. It will be worthwhile to further purify the peptide sequence of royal jelly protein hydrolysates and use clinical or animal models to assess their oxidation inhibition effects.

## Figures and Tables

**Table 1:** Contents of 10-HDA and various flavonoids and phenolic acids in royal jelly protein (RJP) and their hydrolysates.

Sample Species	10-HDA Contents (%)	Flavonoids (mg/100 mg)				Phenolic Acids (mg/100 mg)		
		Quercetin	Naringin	Hesperetin	Galangin	Chlorogenic Acid	Caffeic Acid	Ferulic Acid
RJP *	2.32 ± 0.03 ^c^	16.13 ± 0.06 ^b^	0.47 ± 0.00 ^c^	0.85 ± 0.01 ^c^	0.51 ± 0.05 ^b^	37.61 ± 2.16 ^a^	5.14 ± 0.21 ^a^	68.42 ± 0.25 ^b^
RJP-A	2.74 ± 0.01 ^b^	15.96 ± 0.03 ^b^	0.73 ± 0.02 ^ab^	1.03 ± 0.01 ^a^	0.56 ± 0.02 ^a^	40.33 ± 1.89 ^a^	4.76 ± 0.29 ^a^	72.54 ± 0.14 ^a^
RJP-F	2.68 ± 0.02 ^b^	16.25 ± 0.06 ^b^	0.66 ± 0.01 ^b^	0.87 ± 0.00 ^c^	0.54 ± 0.01 ^ab^	38.26 ± 3.15 ^a^	4.89 ± 0.15 ^a^	74.31 ± 0.22 ^a^
RJP-AF	2.95 ± 0.01 ^a^	18.44 ± 0.05 ^a^	0.76 ± 0.01 ^a^	0.92 ± 0.02 ^b^	0.57 ± 0.02 ^a^	39.68 ± 1.43 ^a^	5.06 ± 0.02 ^a^	73.22 ± 0.17 ^a^

*: RJP: royal jelly protein; RJP-A: royal jelly protein hydrolyzed by alcalase; RJP-F: royal jelly protein hydrolyzed by flavourzyme; RJP-AF: royal jelly protein hydrolyzed by alcalase followed by flavourzyme. ^a–c^: data bearing with identical letter in the same column are not significantly different (*p* < 0.05).

**Table 2:** The contents of free amino acids of RJP and RJP hydrolysates.

Amino Acid (nmol/mL)	RJP	RJP-A	RJP-F	RJP-AF
Aspartic acid	7.84 ± 025 ^d*^	28.47 ± 1.04 ^b^	19.59 ± 0.34 ^c^	36.88 ± 1.25 ^a^
Threonine	3.41 ± 0.03 ^d^	40.52 ± 2.21 ^b^	36.87 ± 0.25 ^c^	46.92 ± 0.68 ^a^
Serine	29.33 ± 0.16 ^c^	196.57 ± 12.41 ^b^	212.67 ± 7.47 ^a^	224.73 ± 3.25 ^a^
Glutamic acid	4.02 ± 0.22 ^d^	80.43 ± 2.88 ^b^	68.59 ± 3.41 ^c^	109.67 ± 3.84 ^a^
Glycine	40.21 ± 0.31 ^c^	358.49 ± 14.32 ^b^	408.72 ± 22.61 ^a^	411.53 ± 18.41 ^a^
Alanine	35.86 ± 0.37 ^c^	573.16 ± 27.47 ^b^	596.42 ± 15.27 ^ab^	643.76 ± 32.77 ^a^
Cysteine	15.92 ± 0.04 ^d^	62.87 ± 3.52 ^a^	48.63 ± 0.54 ^c^	65.58 ± 1.41 ^c^
Valine	15.88 ± 0.19 ^c^	45.16 ± 2.29 ^ab^	42.18 ± 1.36 ^b^	48.27 ± 0.57 ^a^
Methionine	26.74 ± 0.43 ^c^	90.73 ± 11.84 ^b^	116.84 ± 3.57 ^ab^	113.88 ± 4.05 ^a^
Isoleucine	49.53 ± 1.24 ^c^	124.22 ± 12.06 ^a^	103.49 ± 2.95 ^b^	128.49 ± 9.24 ^b^
Leucine	22.84 ± 0.36 ^d^	96.51 ± 2.71 ^a^	78.42 ± 0.67 ^c^	102.48 ± 2.57 ^a^
Tyrosine	12.51 ± 0.06 ^c^	65.33 ± 0.95 ^b^	80.54 ± 0.75 ^a^	81.26 ± 1.84 ^a^
Phenylalanine	7.13 ± 0.15 ^c^	89.29 ± 2.31 ^a^	73.18 ± 3.16 ^b^	93.67 ± 3.16 ^a^
Lysine	55.97 ± 1.52 ^c^	465.82 ± 31.01 ^a^	387.46 ± 22.14 ^b^	487.93 ± 24.19 ^a^
Histidine	16.47 ± 0.01 ^c^	78.69 ± 3.92 ^b^	90.53 ± 4.28 ^a^	94.73 ± 2.53 ^a^
Arginine	24.38 ± 0.31 ^c^	84.93 ± 3.08 ^b^	103.77 ± 3.86 ^a^	105.22 ± 4.17 ^a^
Proine	9.55 ± 0.24 ^c^	92.51 ± 2.72 ^a^	90.19 ± 1.27 ^b^	95.93 ± 3.48 ^a^
EAA **	203.32 ± 4.51 ^c^	1115.87 ± 18.43 ^a^	1032.74 ± 27.41 ^b^	1129.59± 15.76 ^a^

Results from three separate experiments are expressed as mean ± SD. * ^a–d^: data with identical letters in the same column were not significantly different (*p* > 0.05). ** EAA: essential amino acid.

**Table 3:** Effect of royal jelly protein and different hydrolysates obtained by alcalase and flavourzyme on the oxidation of 2′-dG to 8-OH-2′-dG induced by the Fenton reaction.

Addition to RM *	8-OH-2′-dG (μg/mL)			
	RJP	RJP-A	RJP-F	RJP-AF
Blank	0.224 ± 0.02 ^b^	0.224 ± 0.02 ^b^	0.224 ± 0.02 ^b^	0.224 ± 0.02 ^b^
15 mM ascorbic acid	3.410 ± 0.18 ^a^	3.410 ± 0.18 ^a^	3.410 ± 0.18 ^a^	3.410 ± 0.18 ^a^
0.125 mg/mL	0.103 ± 0.002 ^c^	0 ± 0.00 ^c^	0.009 ± 0.00 ^c^	0 ± 0.00 ^c^
0.25 mg/mL	0.080 ± 0.00 ^c^	0 ± 0.00 ^c^	0 ± 0.00 ^c^	0 ± 0.00 ^c^
0.5 mg/mL	0.051 ± 0.01 ^c^	0 ± 0.00 ^c^	0 ± 0.00 ^c^	0 ± 0.00 ^c^
1 mg/mL	0.043 ± 0.01 ^c^	0 ± 0.00 ^c^	0 ± 0.00 ^c^	0 ± 0.00 ^c^
2 mg/mL	0.015 ± 0.00 ^c^	0 ± 0.00 ^c^	0 ± 0.00 ^c^	0 ± 0.00 ^c^
3 mg/mL	0.005 ± 0.00 ^c^	0 ± 0.00 ^c^	0 ± 0.00 ^c^	0 ± 0.00 ^c^
4 mg/mL	0 ± 0.00 ^c^	0 ± 0.00 ^c^	0 ± 0.00 ^c^	0 ± 0.00 ^c^

*: RM (reaction mixture) containing 0.5 mM 2′-dG, 1.3 mM FeCl_2_, 50 mM (H_2_O_2_), 6.5 mM EDTA and 0.1 M phosphate buffer (pH 7.4) was shaken at 37 °C for 30 min. Values with different superscripts were significantly different (*p* < 0.05).

**Table 4:** Effect of royal jelly protein and different hydrolysates obtained by alcalase and flavourzyme on the DNA damage induced by bleomycin-Fe^3+^/Asc and oxidation of 2′-dG to 8-OH-2′-dG induced by Fe^2+^-EDTA/H_2_O_2_/Asc.

Addition to RM *	Bleomycin-Fe^3+^/Asc		Protective Effect of 2′-dG		Fe^2+^-EDTA/H_2_O_2_/Asc	
	Absorbance at 532 nm	Inhibition (%)	8-OH-2′-dG (μg/mL)	Inhibition (%)	8-OH-2′-dG (μg/mL)	Inhibition (%)
Ascorbic acid	0.204 ± 0.01 ^a^ **		7.46 ± 0.16 ^a^ **		10.21 ± 0.32 ^a^ **	
RJP	0.169 ± 0.02 ^b^	17.16 ± 0.24 ^c^	2.86 ± 0.04 ^b^	61.66 ± 0.11 ^c^	6.84 ± 0.03 ^b^	33.01 ± 0.53 ^c^
RJP-A	0.141 ± 0.02 ^bc^	30.88 ± 0.08 ^ab^	1.83 ± 0.06 ^c^	75.47 ± 0.25 ^b^	5.27 ± 0.10 ^b^	48.38 ± 0.74 ^b^
RJP-F	0.153 ± 0.01 ^b^	25.0 ± 0.15 ^b^	1.97 ± 0.03 ^c^	72.23 ± 0.13 ^b^	5.73 ± 0.13 ^b^	43.87 ± 0.23 ^b^
RJP-AF	0.128± 0.02 ^c^	37.25 ± 0.33 ^a^	1.42 ± 0.01 ^d^	80.97 ± 0.34 ^a^	4.57 ± 0.08 ^c^	55.24 ± 1.15 ^a^

* RM (reaction mixture) for DNA damage (containing 0.05 mg/mL bleomycin, 25 μM FeCl_2_, 5 mM MgCl_2_, 0.2 mg/mL calf thymus DNA, 30 mM phosphate buffer (pH 7.4) and 10 μg/mL ascorbic acid) was shaken at 37 °C for 30 min then reacted with 10 μg/mL RJP, RJP-A, RJP-F and RJP-AF for 30 min. ** RM (reaction nmixture) for protective effect and 2′-dG to 8-OH-2′-dG (containing 0.5 mM 2′-dG, 1.3 mM FeCl_2_, 50 mM H_2_O_2_, 6.5 mM EDTA, 15 mM ascorbic acid and 0.1 M phosphate buffer (pH 7.4) was shaken at 37 °C for 30 min. Values with different letters were significantly different (*p* < 0.05).

**Table 5:** Effects of protein and different hydrolysates obtained by alcalase and flavourzyme on the formation of a thiobarbituric acid reactive substance (TBARS) and conjugated dienes on low-density lipoprotein (LDL) oxidation induced by Cu^2+.^

Concentration (mg/mL)	RJP	RJP-A	RJP-F	RJP-AF	RJP	RJP-A	RJP-F	RJP-AF
	TBARS (nmol/mL)				Lag Time (min) *			
Blank	5.41 ± 0.03 ^aA^ **	5.41 ± 0.03 ^aA^	5.41 ± 0.03 ^aA^	5.41 ± 0.03 ^aA^	90	90	90	90
0.01	4.46 ± 0.01 ^bA^	4.41 ± 0.02 ^bB^	4.47 ± 0.02 ^bB^	4.52 ± 0.01 ^bB^	90	120	120	210
0.1	4.14 ± 0.02 ^bA^	4.63 ± 0.01 ^cB^	4.51 ± 0.01 ^cB^	4.74 ± 0.02 ^cB^	120	180	180	270
1.0	3.76 ± 0.03 ^cB^	4.74 ± 0.03 ^dB^	4.79 ± 0.02 ^dB^	4.83 ± 0.01 ^dB^	150	210	210	300

* Conjugated diene formation was measured by determining the absorbance at 234 nm every 30 min for 540 min.** Results from three separate experiments were expressed as mean ± SD. ^A–C^: data with identical letters in the same row were not significantly different (*p* < 0.05). ^a–d^: data with identical letters in the same column were not significantly different (*p*< 0.05).

## Data Availability

Data is contained within the article.
